# Advancing health equity through partnerships of state Medicaid agencies, Medicaid managed care organizations, and health care delivery organizations

**DOI:** 10.3389/fpubh.2023.1104843

**Published:** 2023-03-09

**Authors:** Anna L. Thorndike, Lauren Peterson, Sivan Spitzer, Shilpa Patel, Anne Smithey, Jennifer E. Moore, Scott C Cook, Marshall H. Chin

**Affiliations:** ^1^Pritzker School of Medicine, University of Chicago, Chicago, IL, United States; ^2^Crown Family School of Social Work, Policy, and Practice, University of Chicago, Chicago, IL, United States; ^3^Azrieli Faculty of Medicine, Bar-Ilan University, Ramat Gan, Israel; ^4^Center for Health Care Strategies, Trenton, NJ, United States; ^5^Department of Obstetrics and Gynecology, School of Medicine, University of Michigan, Ann Arbor, MI, United States; ^6^Institute for Medicaid Innovation, Washington, DC, United States; ^7^Department of Medicine, University of Chicago, Chicago, IL, United States

**Keywords:** health equity (MeSH), Medicaid, care delivery transformation, payment reform, cross-sector (social) partnerships

## Abstract

**Background:**

Reducing health inequities in marginalized populations, including people with Medicaid insurance, requires care transformation to address medical and social needs that is supported and incentivized by tailored payment methods. Collaboration across health care stakeholders is essential to overcome health system fragmentation and implement sustainable reform in the United States (U.S.). This paper explores how multi-stakeholder teams operationalized the Roadmap to Advance Health Equity model during early stages of their journey to (a) build cultures of equity and (b) integrate health equity into care transformation and payment reform initiatives.

**Methods:**

Advancing Health Equity: Leading Care, Payment, and Systems Transformation is a national program in the U.S. funded by the Robert Wood Johnson Foundation that brings together multi-stakeholder teams to design and implement initiatives to advance health equity. Each team consisted of representatives from state Medicaid agencies, Medicaid managed care organizations, and health care delivery organizations in seven U.S. states. Between June and September 2021, semi-structured interviews were conducted with representatives (*n* = 23) from all seven teams about experiences implementing the Roadmap to Advance Health Equity model with technical assistance from Advancing Health Equity.

**Results:**

Facilitators of building cultures of equity included (1) build upon preexisting intra-organizational cultures of equity, (2) recruit and promote diverse staff and build an inclusive culture, and (3) train staff on health equity and anti-racism. Teams faced challenges building inter-organizational cultures of equity. Facilitators of identifying a health equity focus area and its root causes included (1) use data to identify a health equity focus and (2) overcome stakeholder assumptions about inequities. Facilitators of implementing care transformation and payment reform included (1) partner with Medicaid members and individual providers and (2) support and incentivize equitable care and outcomes with payment. Facilitators of sustainability planning included (1) identify evidence of improved health equity focus and (2) maintain relationships among stakeholders. Teams faced challenges determining the role of the state Medicaid agency.

**Conclusions:**

Multi-stakeholder teams shared practical strategies for implementing the Roadmap to Advance Health Equity that can inform future efforts to build intra- and inter-organizational cultures of equity and integrate health equity into care delivery and payment systems.

## 1. Introduction

Health equity, where every person has a fair and just opportunity to be as healthy as possible, is increasingly listed as a priority by healthcare organizations and policymakers (1). Nevertheless, health inequities persist across the United States (U.S.) and worldwide. Numerous individual interventions have successfully improved quality of care and health outcomes for people that have been economically and socially marginalized (2). However, widespread scale, spread, and sustainability of health equity interventions requires buy-in and action from all levels of health care organizations, alongside financial incentives and support to enable a viable business model (3, 4). In addition, systemic social drivers of health such as poverty, structural racism and other systems of oppression must be addressed (5).

In the U.S., recent progress to prioritize these efforts has been made at the federal level. For instance, “Advance Health Equity” is one of the five strategic objectives of the Center for Medicare and Medicaid Innovation Strategy Refresh (6). Additionally, the Health Care Payment Learning and Action Network (LAN) Health Equity Advisory Team published a technical guide for advancing health equity through alternative payment models (7). In January 2023, the Centers for Medicare and Medicaid Innovation launched the ACO REACH model, which requires participating accountable care organizations to develop and implement a health equity plan to identify health disparities in their populations and adopt initiatives to reduce these disparities (8). While these changes are promising, efforts to integrate payment reform and care transformation to advance health equity are still in early stages.

COVID-19 inequities and the killings of George Floyd, Breonna Taylor, and others galvanized public awareness and interest in promoting social justice. Health care organizations and policymakers are becoming more aware of the need to address structural racism and the social drivers of health that have led to health inequities. Health care organizations must integrate equity and anti-racism into their internal cultures and design interventions that address racism at institutional, organizational, and interpersonal levels (5, 9). Anti-racism in health care requires shifting power; systemic structural factors driving health inequities must be addressed so that racially minoritized and other marginalized populations can achieve equitable access to care and health outcomes (10). A culture of equity is a culture in which disparities and their causes are openly recognized, staff and providers are motivated to reduce them, and everyone recognizes their role in the process (11). Collaborations across multiple organizations in the health care system must intentionally work to build intra- and inter-organizational cultures of equity and anti-racism to reduce health disparities and advance health equity (12).

In the U.S. health care system, Medicaid is a public insurance program, jointly financed by the federal and state government, that provides coverage to low-income individuals with categorical eligibility. A disproportionately high number of these individuals are people of color (13). The majority of state Medicaid agencies contract with managed care organizations (MCOs) to administer the Medicaid program (14). Clinicians and health care delivery organizations (HCDOs) contract with these MCOs to provide care for individuals with Medicaid coverage. Community-based organizations provide social services and supports to communities, including those enrolled in Medicaid. Thus, collaboration among state Medicaid agencies, Medicaid MCOs, HCDOs, individual providers, Medicaid members, and community-based organizations is essential for sustainable health care reform that advances health equity in the U.S. (15–17).

Advancing Health Equity: Leading Care, Payment, and Systems Transformation (AHE) is a national program supported by the Robert Wood Johnson Foundation that aims to implement strategies to reduce and eliminate health care disparities through care transformation and payment reform. AHE facilitated collaboration among seven multi-stakeholder teams that included representatives from Medicaid agencies, Medicaid MCOs, and HCDOs. AHE supported teams in operationalizing the Roadmap to Advance Health Equity to develop and implement health equity-focused health care initiatives and payment reforms (18, 19). Teams operationalized this model in the contexts of their team dynamics, clinical and community-based settings, and health equity focus. This paper explores how multi-stakeholder teams operationalized and adapted the AHE Roadmap to Advance Health Equity during early stages of their journey to (a) build intra- and inter-organizational cultures of equity and (b) integrate health equity into care transformation and payment reform initiatives.

## 2. Methods

### 2.1. Advancing health equity: Leading care, payment, and systems transformation

In October 2019, the AHE learning collaborative was launched with funding from the Robert Wood Johnson Foundation and comprised of Medicaid stakeholders from seven states: Delaware, Illinois, Maine, New Jersey, Pennsylvania, Tennessee, and Washington. Sites applied to participate in the program and were selected by AHE National Program Office staff. Each state team typically consisted of representatives from the state Medicaid agency, one MCO, and one or more frontline HCDOs. Participating MCOs were contracted with the state Medicaid agency to coordinate and pay for services and supports as part of the Medicaid program. Participating HCDOs were health care delivery organizations that included at least one hospital and physician group. Maine was an exception; the state Medicaid agency does not contract with MCOs but manages care through an accountable care organization for its HCDOs (20). Depending on the state, learning collaborative teams were established from existing informal or formal relationships as well as new relationships between organizations. States received technical assistance from the AHE National Program Office but did not receive any funding for participation other than reimbursement for travel to AHE conferences. Each team worked on their own, met with AHE staff at least monthly for technical assistance on implementing the Roadmap to Advance Health Equity and also participated in cross-team learning collaborative events in which multiple teams received joint training and shared lessons learned. In these individual and group meetings, teams and AHE staff collaborated to identify health equity priorities, perform root cause analyses of these inequities, and design and implement care transformations and payment innovations that address one health inequity experienced by Medicaid members in their settings. One site was an exception; the HCDO included a large network of organizations that were each given the choice to focus on 1 of 3 health equity focus areas selected by the HCDO.

The AHE National Program Office, comprised of individuals from the University of Chicago, Center for Health Care Strategies, and Institute for Medicaid Innovation, provided a didactic curriculum and technical assistance to support teams in implementing health equity initiatives, based on its Roadmap to Advance Health Equity. The Roadmap was developed and updated based upon the team's experience working with grantees implementing equity interventions since 2005 (19, 21), conducting 12 systematic reviews of the health equity intervention literature (19, 22), providing technical assistance to organizations attempting to advance health equity (12), and the University of Chicago's local experience trying to advance diversity, equity, and inclusion (5, 18). The Roadmap has been cited in the National Academy of Sciences, Engineering and Medicine's *System Practices for the Care of Socially At-Risk Populations*, and *The CMS Equity Plan for Improving Quality in Medicare* (23).

**Figure 1:**
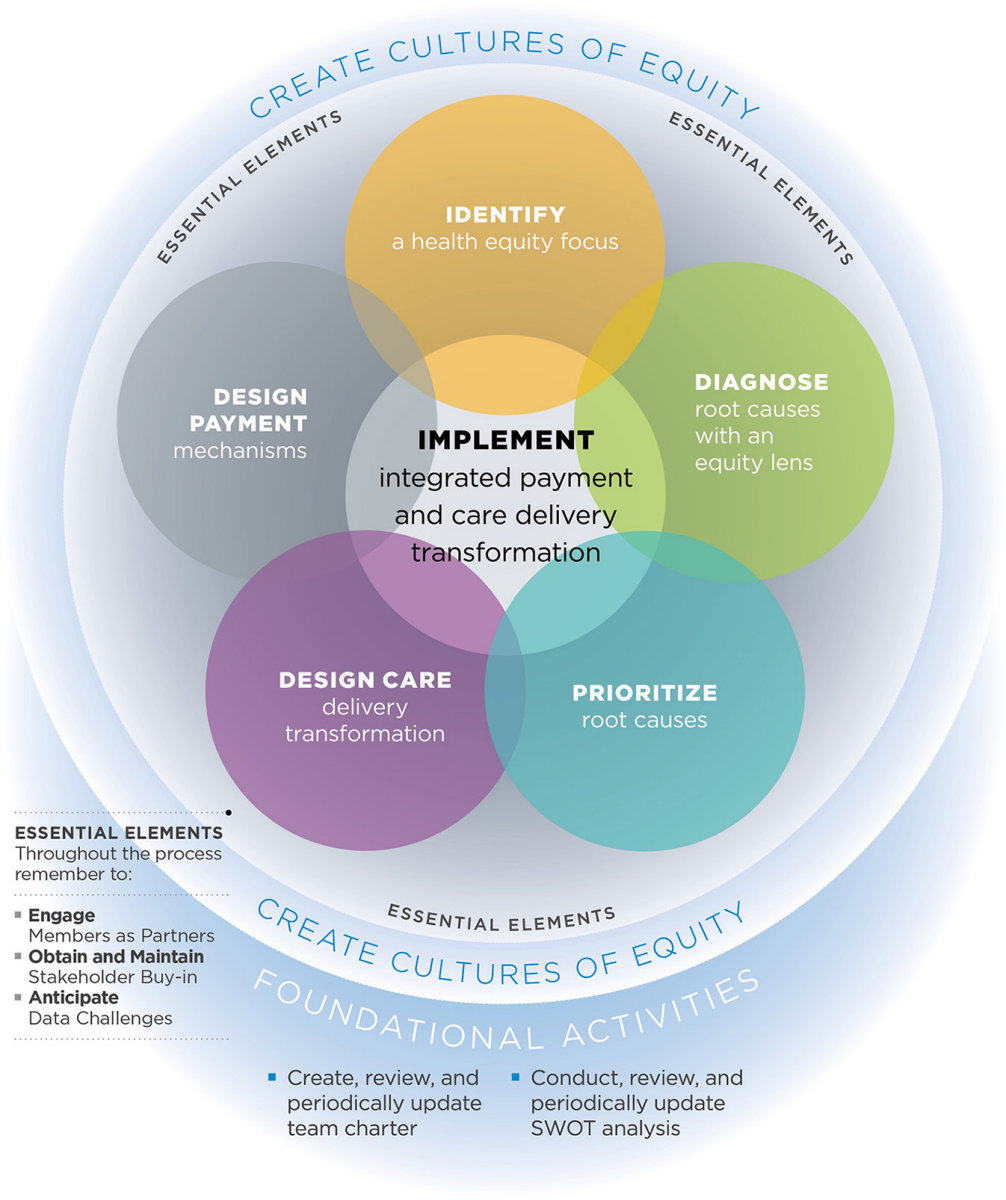
The roadmap to advance health equity^1^.

Roadmap components include (a) creating cultures of equity, (b) identifying a health equity focus, (c) diagnosing root causes with an equity lens, (d) prioritizing root causes, (e) designing care transformation, (f) designing payment mechanisms, and (g) implementing integrated payment and care delivery transformation (Figure 1). The Roadmap also includes Foundational Activities such as creating a team charter and performing a Strengths, Weaknesses, Opportunities, and Threats (SWOT) analysis. Essential Elements of the Roadmap include engaging members as partners, obtaining stakeholder buy-in, and anticipating data challenges (24). Planning for sustainability of the equity interventions was not an independent component, but instead an aspect continuously considered throughout all components of the Roadmap. Each team applied Roadmap components to guide their choice of health equity focus area and implementation strategies into their distinct setting (25). From the beginning of the project, AHE recognized the importance of creating cultures of equity within and among the state Medicaid agency, Medicaid MCO(s), and HCDO(s). In 2020, the COVID-19 pandemic and events of police brutality such as the killings of George Floyd and Breonna Taylor increased societal recognition of the structural racism embedded in the health care system. AHE began encouraging learning collaborative teams to have conversations about how to incorporate anti-racism into their initiatives.

### 2.2. Participants and data collection

Interviews were conducted with learning collaborative team members from state Medicaid agencies, Medicaid MCOs, and HCDOs from each of the seven state sites. Twenty-three semi-structured interviews (4 State Medicaid, 9 Medicaid MCO, 1 Medicaid ACO, 10 HCDO [1 interviewee represented both MCO and HCDO]) took place between June 2021 and September 2021 and focused on the process of intervention design and implementation of the Roadmap to Advance Health Equity as well as building a culture of equity within and across stakeholder groups. All members of state-based teams were invited to participate. Due to competing demands caused by the COVID-19 pandemic, many state Medicaid agency representatives were unable to participate. The interview guide included specific questions about incorporating a culture of equity and conversations about anti-racism into initiative design, team dynamics, the process of designing care transformation and payment reform, and how to achieve sustainability of these reforms (see Supplementary material). Interviews were conducted on Zoom by one white, female University of Chicago doctoral student and lasted approximately 30 to 60 minutes in length. Participants were learning collaborative team members invited to participate by email. Purposive sampling was used with the goal of interviewing at least one team member from each organization. Participants were offered $100 compensation, although nearly all individuals declined compensation because of federal and state laws that prohibit state Medicaid agency and Medicaid MCO staff from accepting compensation or internal policies at their organization. All recruitment materials, oral consent forms, and procedures were approved by the University of Chicago Biological Sciences Division Institutional Review Board (Protocol IRB18-1290). In addition, in May 2021, AHE staff recorded their group impressions of how far each state team had progressed in each step of the Roadmap, categorized as (a) in place or completed, (b) actively working toward completion, or (c) not started.

### 2.3. Data analysis

**Figure 2:**
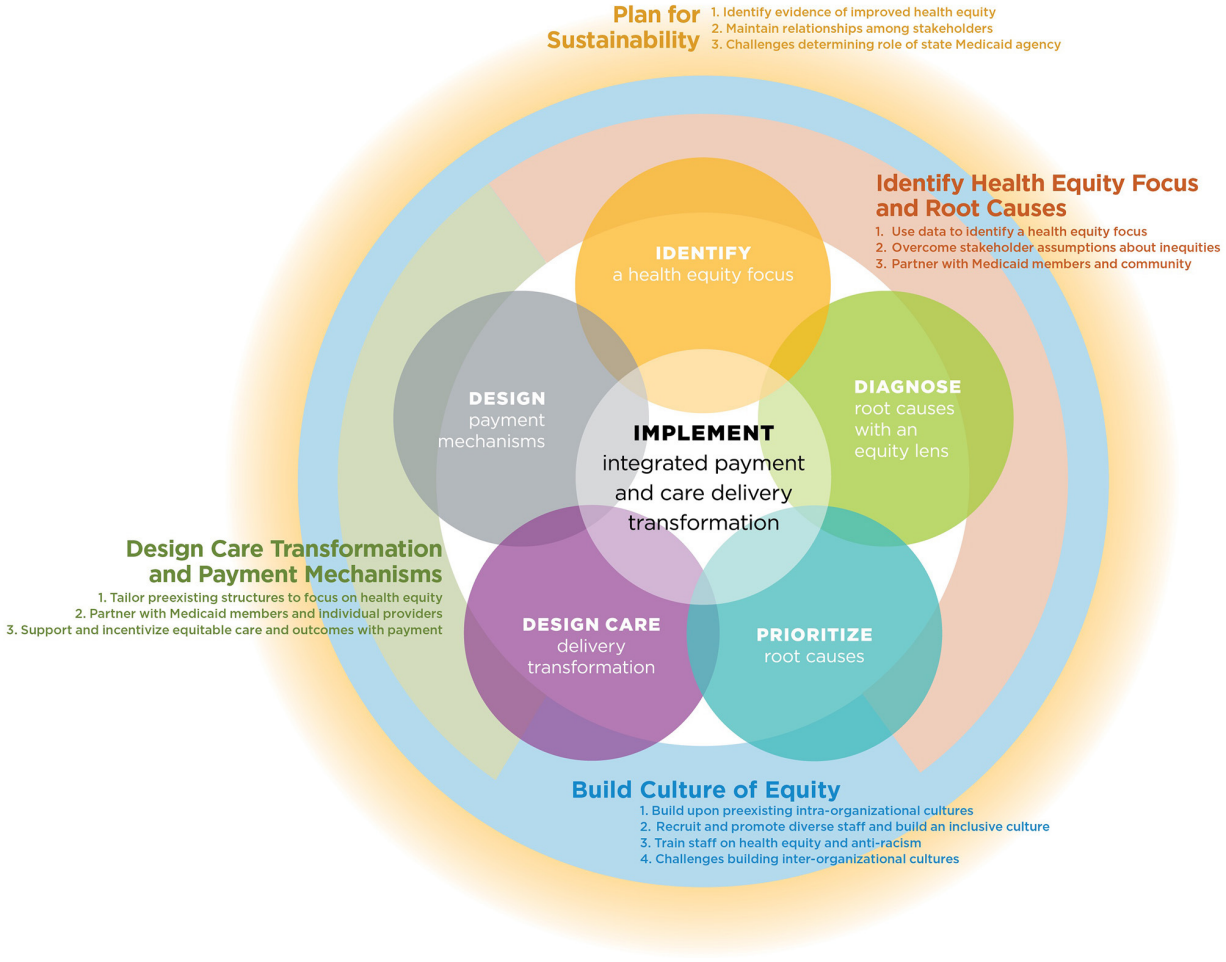
How learning collaborative teams operationalized and adapted AHE roadmap to advance health equity.

The initial codebook was guided by the Roadmap to Advance Health Equity, the Consolidated Framework for Implementation Research, and May's Theory of Implementation (26, 27). NVivo Qualitative Research Data Analysis Software Version 12 was used for coding interview transcripts and thematic analysis. The codebook was developed by eight members of the AHE National Program Office. To pilot the coding process and refine the codebook, two transcripts were coded by each of the eight staff members. Once the codebook and inter-rater reliability was established, two staff members independently coded each transcript in rotating pairs. Staff members met on a regular basis to review code definitions and refine the codebook as needed. Codes were grouped into themes and representative quotes were chosen to illustrate major themes. Figure 2 illustrates facilitators and barriers for each component of the implementation model across the seven teams.

## 3. Results

### 3.1. Participant and learning collaborative characteristics

Table 1 illustrates the health equity focus areas chosen by learning collaborative teams and their progress on the components of the AHE Roadmap to Advance Health Equity. After teams chose their health equity focus area, they performed a root cause analysis of the health inequity, and designed a care delivery transformation and payment mechanisms to address that inequity. Figure 2 depicts how learning collaborative teams operationalized and adapted the AHE Roadmap to Advance Health Equity around four areas of the model: (a) build a culture of equity, (b) identify health equity focus and root causes, (c) design care transformation and payment mechanisms, and (d) plan for sustainability.

**Table 1:** Health equity focus areas of learning collaborative teams and roadmap to advance health equity progress in May 2021^*^.

**Team**	**Health equity focus**	**Identify health equity focus**	**Diagnose root causes**	**Prioritize root causes**	**Design care delivery transformation**	**Design payment mechanism**	**Implementation of interventions**
State 1	Access to health care after release from incarceration or inpatient facility	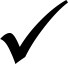	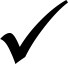	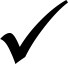	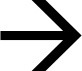	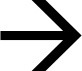	
State 2	Healthcare disparities identified in preventative care and ED over utilization for African American, Black and Hispanic pediatric members.	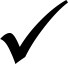	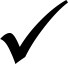	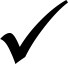			
State 3	Detection and support for high-risk pregnancies in Black perinatal individuals	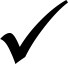	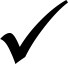	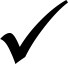	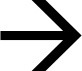		
State 4	1 of 3 areas selected by the HCDO: access to quality health care, pregnancy care, or depression or other chronic condition management	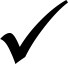	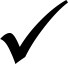	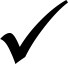	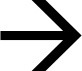	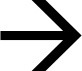	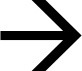
State 5	Housing for families and individuals with serious mental illness or substance use disorder	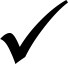	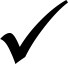	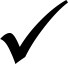	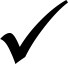	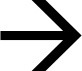	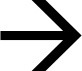
State 6	Sexually transmitted infection (STI) screening and treatment for adolescents of color	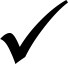	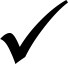	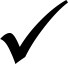	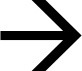	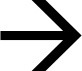	
State 7	Mood disorder diagnosis and treatment in Black postpartum individuals	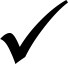	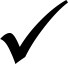	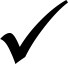	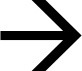		

^*^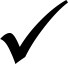, Activity in place or completed; 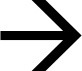, Actively working toward completion of activity; Blank, activity not started.

### 3.2. Build culture of equity

Building a culture of equity within and across the three stakeholder groups was an overarching and ongoing component of the Roadmap to Advance Health Equity. At the opening conference of AHE in October 2019, teams were provided didactic material on how to build a culture of equity within their organization (intra-organizational culture) and across organizations (inter-organizational culture), which was reinforced with subsequent webinars and individualized technical assistance calls over the next 18 months (5, 18). For example, teams received a “Creating a Culture of Equity” strategy overview resource which includes a planning worksheet with categories such as activities, people to engage, sustainability, and a timeline, and a list of external resources.

Thematic qualitative analyses identified three major facilitators to building a culture of equity: (1) build upon preexisting intra-organizational cultures of equity, (2) recruit and promote diverse staff and build an inclusive culture, and (3) train staff on health equity and anti-racism. Analysis also revealed that building inter-organizational cultures of equity was a challenge.

#### 3.2.1. Build upon preexisting intra-organizational cultures of equity

Some organizations joined AHE with existing elements of intra-organizational cultures of equity which accelerated progress. One HCDO representative noted that their organization “has a long and storied history of serving underserved people, [so we] went into this with probably a bigger awareness of systemic racism.” Another HCDO representative described how members of their team had begun prioritizing health equity at their respective organizations prior to joining the learning collaborative, easing the transition. They said, “you look around the room and I often will say this [team] already believes in each other. They already support [health equity] programs.” There was no evidence of preexisting inter-organizational cultures of equity among teams; many relationships between one or more stakeholder groups were new.

#### 3.2.2. Recruit and promote diverse staff and build an inclusive culture

Multiple teams emphasized the importance of recruiting and promoting a racially and ethnically diverse staff within their organization to establish both intra- and inter-organizational cultures of equity. One HCDO representative noted that incorporating more staff from underrepresented populations into the multi-stakeholder team is an area for growth. An HCDO representative from another team described, “we're always thinking about whose voices are missing from the table? Who is underrepresented or who needs to be elevated? We're bringing that equity lens to everything that we do both internally and externally.” Many teams agreed that elevating staff members with identities from underrepresented groups would help create an inclusive culture of equity where people of different backgrounds have a voice in decision-making. In addition, team members highlighted staff who built and advanced a culture of equity by speaking out about anti-racism. One MCO representative said, “Our CEO is vocal, not just with the health equity Office of Diversity and Inclusion and creating that, but regularly sending out things that include [messages about anti-racism].”

#### 3.2.3. Train staff on health equity and anti-racism

Many of the learning collaborative teams also discussed efforts to hire consultants with expertise on equity and anti-racism from outside of their organizations to lead trainings and support the construction of a culture of equity. For example, one MCO representative said, “we need to ensure that if we are going to be operating within this sort of building a culture of equity, that everybody needed a foundational understanding. And so, we have engaged a consulting group to help support around some of that training work.” Staff trainings on equity and anti-racism occurred intra-organizationally within the state Medicaid agency, MCO, or HCDO but not across all organizations.

#### 3.2.4. Challenges building inter-organizational cultures of equity

Many teams struggled to build inter-organizational cultures of equity across sectors. One HCDO representative concluded that intra-organizational cultures of equity and infrastructure must be built before inter-organizational culture can be built:

There was foundational work that needed to be done within each of our organizations before we could get to a place where we could [design payment reform that incentivizes equity work]... I think that the realization that the team came to is there's some cultural change that needs to happen before we can [design payment reform] well.

When asked how the work of creating a culture of equity aligned across the partnering organizations, most interviewees did not describe any concrete action steps that they took to build an inter-organizational culture in the learning collaborative.

### 3.3. Identify health equity focus and root causes

Thematic coding found three major themes of how teams identified a health equity focus and root causes: (1) use data to identify a health equity focus, (2) overcome stakeholder assumptions about inequities, and (3) partner with Medicaid members and community. Participants found this step to be more time consuming than they expected, but it enabled them to integrate equitable processes into their initiative from the outset.

#### 3.3.1. Use data to identify a health equity focus

Teams differed on their approach to using data to identify disparities and root causes. Teams investigated inequities in health care and outcomes from data sources including claims records, electronic health records, and state Medicaid population data. Team members agreed that data were essential to identifying a health equity focus, but sometimes disagreed about the most useful way to incorporate and analyze data. One state Medicaid representative expressed the value of data, “we had a group look at some claims data in Medicaid and do a pretty comprehensive analysis of some discrete health outcomes related to certain demographic characteristics... We looked based on race, ethnicity, I think we did language, we've done geographic region, sex, age.” In contrast, an MCO representative from another state believed that looking at quantitative data within the Medicaid population neglected to identify additional health inequities that exist between the Medicaid population and the general population which might be better captured with qualitative data:

I think [when] we're looking at a Medicaid population specifically, we don't see the variances... when you start to dissect the data so much and you're looking at only one provider and only one population... you may not see some of the food insecurity that we know it's not, that it's not jumping out in the data because you're looking at such small numbers. But we know that in talking to the community that that's an issue.

One state Medicaid representative from another team found that a lack of available data on health disparities made it difficult to identify an inequity to focus on in their community, “what happens is that when we go back and look at our data... we obviously know where disparities exist, but we don't have a lot of data to support that in some ways.”

#### 3.3.2. Overcome stakeholder assumptions about inequities

Some teams had to challenge learning collaborative team members' preexisting ideas of which health disparities were most prevalent in and important to their member population. Often, these assumptions did not necessarily match what the data or Medicaid member engagement revealed to be their most prevalent or salient health inequities. For example, one HCDO representative explained, “we had a [health equity focus] in mind and we went to the community and they said this isn't actually what we want to work on. So, we had to do an about face and [the MCO] was very flexible.” Participants expressed that overcoming these stakeholder assumptions was a critical step to identify the health inequity and subsequently design the initiative.

#### 3.3.3. Partner with Medicaid members and community

Many teams partnered with Medicaid members and other community members through interviews, focus groups, or surveys to help identify a health equity focus. For example, one state Medicaid representative highlighted the usefulness of soliciting feedback directly from community members before choosing a health equity focus, “we had focus group meetings earlier on with those that were in the state jail... We used the focus group in order to diagnose the disparities.” An MCO representative from a different state expressed a desire to continue incorporating member feedback in the future, “I think we'll take back a lot of [lessons about] how to engage with our members. We built our root cause analysis and we created a [Medicaid] member survey so we could kind of get some more member feedback.”

### 3.4. Design care transformation and payment mechanisms

After identifying the health inequity and conducting a root cause analysis to identify where the team could focus their efforts, teams began to design and implement the care transformation and payment reform components of the Roadmap to Advance Health Equity. Implementation themes included: (1) tailor preexisting care delivery and payment structures to focus on health equity, (2) partner with Medicaid members and individual providers, and (3) support and incentivize equitable care and outcomes with payment.

#### 3.4.1. Tailor preexisting care delivery and payment structures to focus on health equity

Participants from three teams shared the advantages of incorporating new health equity measures into existing payment plans. For example, one MCO representative said:

We have been lucky because we already had a program in place that was paying for value instead of fee-for-service models... we're not building something brand new. Instead, how do we take something that already has years of history and has been working for us and just add an equity component.

An HCDO representative from another team described the usefulness of existing payment models, “if you're already in a value-based payment methodology with that payer, which many of the health systems are now, just pick metrics that you already have and that you've already been working on and then add the disparities lens to it.”

#### 3.4.2. Partner with Medicaid members and individual providers

Many teams described the importance of incorporating Medicaid member engagement into the implementation of care transformation and payment reform process steps. For example, one HCDO representative shared their experience of implementing member feedback:

Really getting input from people who have experienced homelessness. So, we have a lived experience advisory committee and they've been really integral in asking us really big questions that we didn't think about as staff. So, I think it's really important to have that feedback and input throughout the entire process.

An HCDO representative from a different state similarly expressed a desire to “try whenever possible to include the community in the design process.” Some teams also highlighted the importance of soliciting feedback from providers about the feasibility and efficacy of the care transformation, since limitations such as provider time and workflow can pose barriers to incorporating new care transformations.

#### 3.4.3. Support and incentivize equitable care and outcomes with payment

Teams described two approaches to support and incentivize equitable care and outcomes with payment.

##### 3.4.3.1. Identify and implement health equity metrics

Teams sought metrics that could capture improvements in their health equity focus area over time. One MCO representative said, “I hope that more dollars are tied to very specific health equity outcomes.” Additionally, one HCDO representative from a different state proposed using lab values such as hypertension and diabetes markers to study changes in the health equity focus area:

I think if this pilot initiative results in significant reductions of those aforementioned lab values for the different diagnoses—hypertension, diabetes, drug addiction, et cetera—I think more providers will be incentivized to join the initiative or participate in initiative. The state will be incentivized because they'll see the cost savings in the plans.

Some team members recommended incentives for health improvements specific to the population with the highest risk of reduced health outcomes. For example, one MCO representative said, “We're looking at improvements with the prioritized populations separately than the population at whole and creating an added incentive for those improvements to acknowledge that it may be more difficult to reach with the prioritized population because there are more barriers.”

##### 3.4.3.2. Reimburse for new activities

An MCO representative from a different state introduced the idea of incentivizing health equity activities by reimbursing for preventative services related to the health equity focus area, such as screening patients for sexually transmitted infections or mood disorders, saying, “thinking through the pay[ment] model so that we can be reimbursed for preventative services or screenings and not reimbursed for things that go wrong, as we want to better this pay model and really want to look toward value-based care and recognizing the patient as a whole.” At the time the interviews were conducted, most teams were in the midst of planning their payment reform strategies and had not implemented these methods.

### 3.5. Plan for sustainability

Teams described planning for program sustainability as an ongoing challenge and critical component of the implementation process. The major facilitators of teams' approach to sustainability were: (1) identify evidence of improved health equity in area of focus and (2) maintain relationships among stakeholders. One challenge of sustainability was determining the role of the state Medicaid agency.

#### 3.5.1. Identify evidence of improved health equity in area of focus

Many team members described that the financial sustainability of their care transformation depended on evidence that their initiative improved health equity. One state Medicaid representative said, “I think if we can show that we can move the needle in addressing some of the disparities that were identified... we're then able to scale this and we're able to ensure sustainability.” Proof of improved experiences and outcomes for members that face health inequities could convince stakeholders to make a future investment. An HCDO representative from a different state described:

I think the primary stakeholders want to see an immediate return on their investment. And a return on investment is that data that shows that those values that I talked about have decreased and outcomes and patients are healthier. I think that will be the catalyst for sustainability.

#### 3.5.2. Maintain relationships among stakeholders

Long term relationships among state Medicaid agency, Medicaid MCO, and HCDO representatives were described as the foundation of their care transformation, and therefore essential to its long-term success and survival. One HCDO representative said:

It's not locked-in sustainability. But to try to get three large organizations just talking about a complicated topic like this more than once is helpful in terms of sustainability. And I think we all kind of agreed that we would be better off working together than working in silos.

Some team members also highlighted that maintaining close relationships with individual providers in particular would promote sustainability since providers can help advocate to organizational leadership that the care transformation is worth investing in long-term. For example, an interview with an HCDO representative from the same state noted, “the providers have expressed a desire to continue the funding because it helps them sort of make the case with leadership to be able to focus on this kind of work... So that feedback has been helpful to already start that conversation about keeping the investments going.”

#### 3.5.3. Challenges determining role of state Medicaid agency

Teams provided mixed feedback on what the role and level of engagement of the state Medicaid agency would be in the future for sustainability of the care transformation and payment reform. In two states, team members expressed that their partnership with the state agency might be challenging in the long term. One MCO representative said:

Probably the connection that was created with the state, just because I don't feel as though, at least in [state X], that they've really stepped up or even come to the table as much as the provider and the payer. So, I would envision potentially the provider, [MCO], and [HCDO] continuing down this road together, but I'm not so sure the state would continue to be a stakeholder, even though ultimately they have the most power.

However, another state's team members disagreed. They foresaw the state Medicaid agency playing a critical role in the sustainability of their program. One MCO representative stated, “as part of value-based partnerships in general, I would say the greatest potential lies in continuing to work with the state, as the state has the reach over the whole Medicaid population.”

## 4. Discussion

Learning collaborative teams shared many themes about how they built intra-organizational cultures of equity and adapted their approaches to the AHE Roadmap to Advance Health Equity. As expected by the Roadmap, teams' approaches varied due to their different contexts, such as existing infrastructure and culture, team dynamics, prior relationships with partners, and health equity focus areas. Teams worked to reach consensus across the three stakeholders during each component of the Roadmap to Advance Health Equity. Our qualitative approach enabled us to gather practical, frontline examples of how health equity goals are operationalized into action steps (28).

### 4.1. Build culture of equity

Teams reported that building a culture of equity and anti-racism was facilitated by hiring a diverse staff and impacted by teams' abilities to hire, promote, or support staff. Our findings support prior evidence that successfully using equity frameworks requires specific guidance for implementing organizational change (4), while additionally offering insights into the role of context and the practical realities of implementation. For example, some teams were able to hire new staff members in diversity, equity, and inclusion roles. Other teams used alternate strategies such as existing leadership speaking out about anti-racism to build a more inclusive culture. Our finding that staff training supported building a culture of equity and anti-racism aligned with prior evidence on the value of training a core group on equitable implementation practices (29).

Teams had greater success developing intra-organizational cultures within their separate organizations than creating a shared, inter-organizational culture across stakeholders. Some teams felt it was important to create an intra-organizational culture of equity before the inter-organizational culture. Efforts to build a shared inter-organizational culture were at earlier stages and might benefit from more time and additional technical assistance. In addition, events of 2020 that highlighted structural racism, such as the COVID-19 pandemic and police brutality toward people of color, occurred soon after the launch of the AHE learning collaborative. Thus, the learning collaborative's technical assistance around discussing and addressing structural racism has evolved. Learning collaborative teams will benefit from the provision of (1) clear definitions and examples of cultures of equity, (2) action steps for how to build a culture of equity across organizations, especially organizations from different sectors of the health care system, and (3) guidance on creating intra- and inter-organizational cultures of anti-racism (5, 18).

### 4.2. Identify health equity focus and root causes

Identifying a health equity focus and diagnosing and prioritizing root causes of health inequities are key components of the Roadmap to Advance Health Equity. Many teams faced the unexpected barrier of overcoming stakeholder bias and assumptions about which health disparities were most widespread and pertinent among their members. Teams reported that a combination of using data to identify disparities and Medicaid and community member engagement, input, and feedback were the best facilitators for accurately identifying a health equity focus area that was truly important and valued by all stakeholders. Future teams would benefit from partnering with Medicaid members to improve initiative design and implementation throughout all Roadmap components. Combining use of data and Medicaid member feedback with implicit bias training could help identify and reduce inappropriate stakeholder assumptions (30).

### 4.3. Design care transformation and payment mechanisms

Teams developed different strategies for incentivizing health equity in their care transformations and payment reforms. Previous research has established the value of tying equity performance measures to payment incentives to reduce disparities (31). While all learning collaborative teams agreed on the importance of this coupling, our findings also demonstrate the challenge of identifying and selecting appropriate methods to measure improved health equity. Teams also expressed the value of building upon existing payment models. While it may be easier to build upon existing concepts and infrastructure, it is also critical to explore the universe of different payment levers for the combinations that could provide the most effective vehicles for advancing health equity in different contexts. For example, upfront payments for equity- promoting infrastructure and services such as information systems with medical and social needs data, community health workers, and outreach teams for preventative services; incentive payments for reducing inequities; and risk-adjusting payment for social risk could all help advance health equity (7, 32–34). In addition, it is important to apply specific health equity and anti-racist approaches to these payment methods (35).

### 4.4. Plan for sustainability

Teams grappled with sustainability of their partnerships and interventions. Many participants believed that maintaining strong relationships among the stakeholder groups was essential to sustainability. The intensity of state Medicaid agency participation varied across teams. Responsive, engaged state agencies are critical to the success of health equity initiatives for individuals with Medicaid insurance, contributing to program effectiveness and sustainability (36). Future contracts between state Medicaid agencies, managed care organizations, and HCDOs should support and incentivize meaningful inter-organizational collaboration to advance health equity and sustain initiatives.

### 4.5. Limitations

Our study has several limitations. First, this was an exploratory study aimed to capture the perspectives of stakeholders at an early stage of the implementation process. Teams were still in the process of designing and implementing their care transformations and payment reforms at the time of the interviews, and thus team members could not provide information on a complete process of implementation. Second, some learning collaborative teams and stakeholders had limited representation among the interviewees, and only those team members most closely engaged with the project were invited to participate in this round of interviews. However, we were able to interview some team members from all seven state teams and across all three types of stakeholders. Third, quantitative methods such as frequency counts of themes were not used to analyze this small qualitative dataset as some researchers do for larger, more representative qualitative datasets. Themes will need to be confirmed in future studies. Fourth, the AHE project took place during the COVID-19 pandemic, which may have slowed teams' progress in implementation and integrating goals of health equity and anti-racism. Alternatively, the COVID-19 pandemic and the sociopolitical conditions of 2020 and 2021 may have created increased momentum for health equity work. Fifth, at the time (May 2021) the AHE staff provided their perceptions of what stage each team was at in the Roadmap, we did not verify these impressions with the state teams.

### 4.6. Conclusions

Teams comprised of multiple stakeholder groups grappled with common themes as they built cultures of equity and operationalized and adapted the AHE Roadmap to Advance Health Equity. Collaboration among state Medicaid agencies, Medicaid MCOs, and HCDOs to implement payment reform that supports and incentivizes care transformation to advance health equity was possible and productive. Stakeholders worked continuously to align their goals across organizations, develop a shared culture of equity, and integrate equity and anti-racism into actionable initiatives. Teams adapted their implementation approaches to their contexts, including settings, team dynamics, and health disparities of focus. Future directions could include examining teams when they are further along in the process of implementation, using both quantitative scales and qualitative methods to assess participants' perceptions of their teams' progress implementing the Roadmap to Advance Health Equity, and analyzing whether different stakeholders vary in their perceptions and experiences in the initiative. We also plan to do more case studies as we have done in collaboration with the Illinois state team (25). Additional guidance to multi-stakeholder teams on how to take collaborative inter-organizational actions to build shared cultures of equity and anti-racism and intentionally transform care delivery and reform payment to advance health equity could reduce longstanding health inequities in the U.S. and globally.

## Data availability statement

The raw data supporting the conclusions of this article are unavailable to protect the confidentiality of the participants.

## Ethics statement

The studies involving human participants were reviewed and approved by University of Chicago Biological Sciences Division Institutional Review Board. The Ethics Committee waived the requirement of written informed consent for participation.

## Author contributions

AT, LP, SS, and MC conceived the study scope and aims. JM, LP, MC, SP, and SS helped develop the interview questions. AT, JM, LP, MC, SP, SS, and AS participated in data interpretation. AT, LP, SS, SP, AS, JM, SC, and MC were involved in drafting and revising the manuscript. All authors contributed to the manuscript and approved the manuscript for publication.

## Footnotes

^1^Copyright 2020 by Advancing Health Equity: Leading Care, Payment, and Systems Transformation, a program funded by the Robert Wood Johnson Foundation.
