# Supplementary material for: Advancing health equity through partnerships of state Medicaid agencies, Medicaid managed care organizations, and health care delivery organizations
Source: Front Public Health. 2023 Mar 9;11:1104843. doi: 10.3389/fpubh.2023.1104843 (PMC10035573; doi:10.3389/fpubh.2023.1104843)
Supplement: Supplementary file 1 [file Data_Sheet_1.docx]

**2021 Advancing Health Equity Qualitative Interview Questions**

1. Please tell me a little bit about your role in the Advancing Health Equity Initiative.
2. What do you think about the project?
   1. What have been (were) some of the most significant challenges your team has faced?
   2. What have been the biggest takeaways from being part of the AHE initiative?

*Team & Project Dynamic Probes*

1. How does your team work together (e.g. what work processes has your team created to work together)?
   1. Probe: Have relationships between organizations changed at all? And if so, how?
   2. Probe: Have relationships between people within your organization change at all? If so how?
2. Since the Learning Collaborative launched in 2019, what changes have been made or are currently being considered at your organization or partnering organizations as a result of the AHE initiative?
3. How has your context such as social, political, or organizational factors impeded or accelerated your work?

Probes: For example, racial justice movement, your organization’s view of equity

*Culture of Equity Probes*

1. What does a culture of equity mean to you?
2. How is equity part of your daily work, if at all?
3. *If applicable:* How is your organization establishing a culture of equity?
   1. Probe: Are these efforts new since the launch of the AHE in 2019 or has there been work in this space prior?
   2. Probe: How has this work aligned across the partnering organizations?
   3. Probe: How is your organization engaging in anti-racism conversations and/or work?

*Care Delivery Transformation Probes*

1. What has worked well or is working well as your team is designing your care delivery reform?
   1. What aspects of designing care delivery reform were challenging or surprising?
   2. What were successful strategies to overcome those challenges?
   3. How has incorporating a health equity lens changed how your team has approached designing a care delivery transformation?

*Payment Reform Probes*

1. What has worked well or is working well as your team is designing a payment reform?
   1. What aspects of designing a payment reform were challenging or surprising?
      1. What were successful strategies to overcome those challenges?
      2. How has incorporating a health equity lens changed how your team has approached designing a payment reform?
2. Are there additional ways, not already discussed, that states or payers could incentivize or encourage health care organizations to prioritize health disparities reduction?
   1. Given everything that the partner organizations must attend to, what would have to happen for them to prioritize health disparities reduction and elimination?
   2. Are there aspects that make it more difficult to change how care is contracted or paid for as a way to reduce disparities?
      1. Probe: CMS Guidance, State budget constraints, requirement for actuarial soundness of payment to MCO’s, unknown costs of specific interventions or payment models

*Sustainability Probes*

1. What are your overall plans for sustaining your interventions developed through the Learning Collaborative?
   1. Probe: are there plans to continue other aspects of the learning collaborative efforts (e.g. improving data reporting capabilities, continue building relationships across partner organizations, continue building cultures of equity)
   2. Probe: Is there anything that you anticipate may be discontinued after the end of the formal initiative due to insufficient financial resources, time, etc.?
2. What particular outcomes could help to make a case for future investment? (*e.g., for providers-- clinical outcomes, utilization, patient experience or engagement, staff retention, staff burnout; for states and plans-- ROI, infrastructure payments for financial resources*)

*Closing Questions*

1. What you’re trying to do is extremely hard and time intensive. What are you most proud of as a result of the AHE initiative?
2. If you were to offer tips to other [*organizations* e.g. MCOs] hoping to align themselves with [*partner organizations* e.g. frontline providers & states], what would they be?
   1. Probe: Looking back from where you are now, is there anything you would have done differently?
3. Is there anything I did not ask, that you think is important to know?
